# The influence of curvature on the properties of the plasma membrane. Insights from atomistic molecular dynamics simulations

**DOI:** 10.1038/s41598-017-16450-x

**Published:** 2017-11-22

**Authors:** Semen O. Yesylevskyy, Timothée Rivel, Christophe Ramseyer

**Affiliations:** 1 Department of Physics of Biological Systems, Institute of Physics of the National Academy of Sciences of Ukraine, Prospect Nauky 46, Kyiv, 03680 Ukraine; 2 Laboratoire Chrono Environnement UMR CNRS 6249, Université de Bourgogne Franche-Comté, 16 route de Gray, 25030 Besançon, Cedex France

## Abstract

In this work we conduct a systematic analysis of the influence of curvature on various properties of a realistic model of mammalian plasma membrane with asymmetric lipid content of monolayers and a realistic concentration of cholesterol. In order to do this we developed new technique for simulating membranes with the global membrane curvature restricted to any desirable value while keeping free lateral diffusion of the lipids and without introducing artifacts or perturbing the membrane structure. We show that the thickness of the hydrophobic core of the concave monolayer decreases by approximately 1.3 Å in comparison to that of the flat membrane, while the thickness of the convex monolayer does not change. The order parameter of the lipid tails decreases significantly in the certain layers of the curved membrane. The area per lipid increases in the convex monolayer and decreases in the concave monolayer. The cholesterol inclination angle decreases when the curvature of a particular monolayer changes from concave to convex. The amount of cholesterol in the minor fraction located between the membrane leaflets is zero in the membrane with positive curvature and increases to 1.7% in the flat membrane and to 2.5% in the membrane with negative curvature.

## Introduction

There is a variety of membranes in the cell which can act as physical barriers that both separate the inside of the cell from the external medium, and facilitate compartmentalization and specialization of the intracellular medium. Cellular membranes are highly dynamic assemblies, changing their shape, composition and properties on a variety of spatial and temporal scales. The reshaping of membranes occurs during different cell processes such as motion, division, differentiation and vesicle trafficking. Membranes must be sufficiently rigid and robust to maintain the integrity of the cell compartments and flexible enough to allow continuous changes of their shapes.

Recent studies have shown that membrane curvature is not a passive consequence of cellular architecture but an active driving force in many processes involving membrane remodeling and trafficking^1^. Local differences in membrane curvature form microenvironments in which specific molecular interactions are more likely to occur. Formation of the membrane curvature is achieved by the complex interplay between intrinsic curvatures of the lipids in the membrane leaflets, the influence of integral and peripheral proteins and the influence of the cytoskeleton. While the intrinsic spontaneous curvature of the membrane is mostly determined by its lipid composition, instantaneous local curvature is usually dynamically modulated by proteins which are either integrated into the lipid bilayer thus acting like wedges or attached to its periphery. In eukaryotes, the Bin/Amphiphysin/Rvs (BAR)-domains containing proteins^2^ are well-known for their ability to form 3D assemblies and act as molecular scaffolds that reshape the membrane and alter its mechanical properties. While the membrane proteins and the influence of the cytoskeleton could lead to dramatic changes in the membrane shape and curvature, the membrane lipids should redistribute and repack to adapt to these changes. Surprisingly, little attention is paid to lipid repacking and redistribution in curved membranes despite the fact that changes in the lipid composition are known to be important for a range of pathologies, such as cancer^3^, Alzheimer’ s disease^4^ and obesity^5^.

The asymmetry of the membrane leaflets is another important feature of eukaryotic membranes which is closely related to the membrane curvature and shape. It influences the mechanical properties of the membranes and their passive permeability to various compounds. It is well known that phosphatidylcholine (PC) and sphyngomielin (SM) are located mostly in the outer leaflet of the plasma membrane, while phosphatidylethanolamine (PE), phophatidylserine (PS) and phosphoinositides are abundant in the inner leaflet^6,7^. This distribution is maintained by active energy-dependent transport of lipids and is vital for the functioning of normal cells. The asymmetry disappears in the course of apoptosis and in many types of cancer cells, which makes it one of prospective markers of apoptosis and cell malignancy^8–10^.

In addition to the asymmetry of lipids, the distribution of sterols, which are the most abundant molecules in eukaryotic membranes after the lipids, is also remarkably uneven^11–13^. Although existing experimental techniques cannot reliably determine the distribution of cholesterol in the asymmetric membranes^14,15^, an important role of cholesterol in controlling the membrane curvature and asymmetry is well established. Cellular phenomena such as the formation of synaptic vesicles^16^ and apoptotic bodies^17,18^, the membrane fusion^19,20^, budding of enveloped viruses from the plasma membrane^21,22^, formation of the blebs during apoptosis^17,18^, blood cell maturing^23^ and mitosis^24^ are likely to be influenced by phospholipid asymmetry, membrane curvature and cholesterol distribution^25^.

Curvature, asymmetry, lateral heterogeneity and non-trivial dynamic properties make real cell membranes very complex objects, thus making them hard to study experimentally with nanoscopic resolution. Such complexity is behind the rapid progress in computer simulations of realistic cell membranes. These *in silico* techniques are nowadays considered as the best complementary techniques in membrane science since they are able to operate at an atomistic level. There are a significant number of computer simulation studies which deal with multicomponent membranes of different sizes and complexity in particularly, the plasma membranes of normal and cancer cells (such as thymocytes, hepatocytes and their derived malignant cells) were simulated in all atom details recently^26,27^, however, the leaflets of the membranes were considered symmetric in this study. The difficulties of experimental studies of cholesterol partitioning and dynamics in lipid bilayers have also stimulated numerous *in silico* studies of these phenomena^28–34^.

With the development of high-quality coarse-grained (CG) methods, such as MARTINI force field^30–33^, the global trend of membrane simulations shifted away from atomistic simulations. Coarse-grained models allow increasing time and length scale of simulations by several orders of magnitude. In the CG models the groups of adjacent atoms are combined into “beads” which interact with each other by means of empirical potentials. Since the number of beads is much smaller than the number of individual atoms, a significant increase in the speed of computations could be achieved^30–33^. In particular, CG methodology allows the simulation of very large membrane patches with a large number of lipid species, and which could be considered as accurate models for real cell membranes in terms of lipid content^35^. However, this study was focused on a flat membrane. Despite impressive progress in the field of CG simulations all atom molecular dynamics studies of the membranes are still necessary to achieve an accurate atomistic description. Notably, atomistic simulations are necessary in the studies of detailed interactions of ligands and drugs with the membranes. An atomistic description also provides the most detailed view on the effects of curvature and asymmetry on the membrane properties including the translocation of ligands and drugs through the lipid bilayer.

There are few studies dedicated to atomistic simulations of the effects of curvature in realistic asymmetric cell membranes. To the best of our knowledge there are no comprehensive and systematic studies which address the influence of curvature on the realistic membrane with asymmetric lipid content of monolayers. One of the possible reasons for this is the methodological difficulty of setting up and running such simulations. Indeed, the membrane curvature is a transient property of the membrane which changes from one region to the other and evolves quickly over time^36^. In order to study the changes of various membrane properties as a function of curvature, one needs either to fix the curvature or to sample only those regions of the membrane which display the needed curvature at each simulation time step. The second option seems to be more attractive since it does not impose artificial constraints on the membrane and the methods for determining both local^37^ and global^38^ membrane curvatures as a function of atomic coordinates are available. However, sampling a sufficiently wide range of curvatures in free all atom MD simulations is problematic because of the required simulation times (up to hundreds of microseconds). Such times are accessible only in a few individual state-of-the-art simulations but they are still prohibitively large for routine use. In contrast, restricting global membrane curvature to a particular predefined value is much more promising in this respect since it makes the problem much more computationally viable. However, no technique for restricting membrane curvature to some given value is available to date.

The present work introduces a new technique for simulating membranes with pre-defined curvature which is applicable at both the atomistic and coarse grained levels. The essence of our technique is applying repulsive walls of dummy particles, which only interact with hydrophobic core of the membrane and maintain any desired curvature while keeping free lateral diffusion of the lipids and without introducing artifacts. Our technique does not change the basic properties of the membrane such as density, thickness or area per lipid and allows studying the influence of the membrane curvature itself on these parameters.

Using this technique, we present a systematic analysis of the influence of curvature on various properties of a realistic model of mammalian plasma membrane with asymmetric lipid content of monolayers and a realistic concentration of cholesterol. We revealed several non-trivial effects of curvature, such as compression of the hydrophobic core of concave monolayers, a significant decrease in the ordering of lipid tails in certain layers of the membrane, systematic changes of the areas per lipid and the angle of inclination for the cholesterol, etc. To our knowledge our study is the first successful attempt to reveal such fine-grained details concerning the influence of curvature in realistic all atom models of the membranes.

## Results and Discussion

### The influence of the walls

**Figure 1:**
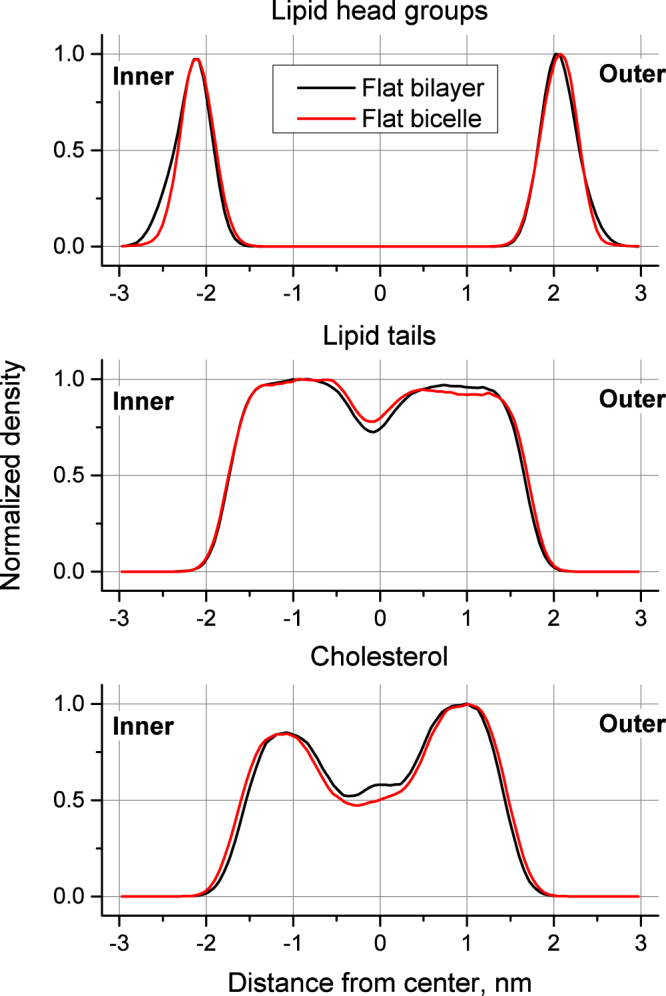
Normalized densities of lipid head groups, lipid tails and cholesterol in reference flat bilayer and flat bicelle of the same composition.

In order to check whether the repulsive walls, which are used to maintain the membrane curvature (see Methods for details), influence the structure of the membrane, we prepared a reference bilayer, which is free from any restraints and has the same lipid content as the flat part of the bicelle with restricting walls. We used the last 50 ns of the trajectory of equilibrated flat bicelle and counted the mean number of lipids and cholesterol in its bilayer region excluding the caps. We then constructed the flat infinite bilayer with the same composition and simulated it using the usual setup with semi-isotropic pressure coupling for 200 ns without any restraints. We then computed the density profiles for the lipid heads, lipid tails and cholesterol molecules across the membranes in the cases of flat reference bilayer and the bicelle with zero curvature. The results are shown in Fig. 1.

It can be clearly seen that the influence of the walls on the distribution of lipid head groups is minor. The walls slightly decrease the outer parts of the distribution for head groups in the region around 2.5 nm from the center. Thus the walls marginally decrease the number of headgroups protruding far into the bulk water while keeping their average position and other parts of distribution unchanged. The distributions of the lipid tails with and without the walls are almost indistinguishable except for the central region where minor deviations are observed. The thickness of the hydrophobic membrane core is not affected by the walls. The distribution of cholesterol is marginally broader in the bicelle with the walls, but this effect is very small. Differences in the central region of the membrane are rather chaotic and are likely to be caused by an undersampling of cholesterol diffusive motions, which is inevitable in such a complex multi-component system.

In order to verify if the influence of repulsive walls depends on lipid composition we additionally studied simple monocomponent membrane composed from pure 1,2-dioleoyl-sn-glycero-3-phosphocholine (PC). For this membrane we prepared the reference bilayer without restrictive walls and the planar bicelle with the walls in the same way as for asymmetric membrane, described above. We then computed the density profiles for the lipid head groups and tails in both systems. The results are shown in Supplementary Fig. S1 available online. They are very similar to Fig. 1, which confirms that the influence of the walls remains minor regardless of the lipid composition of the membrane.

It is possible to conclude that the repulsive walls in our setup have almost no influence on the major structural properties of the flat membrane such as the thickness of the hydrophobic core, average location of the head groups and distribution of cholesterol. In the case of the curved bicelles it is not possible to separate the influence of the walls from the influence of the curvature itself because the walls are necessary to maintain the curvature. We assume that the effects observed in the curved bicelles are caused mostly by the membrane curvature itself while the influence of the walls remains as small as in the flat system. Such extrapolation may not be completely accurate but, given the absence of any reference system for curved membranes, it remains the best possible assumption.

### Influence of the curvature on density profiles of membrane components

The density profiles of the different chemical groups of lipids are the most obvious indicator of the membrane structural changes upon bending. These profiles are very well known for flat model membranes from both experiments and simulations. Thus, it is interesting to see if the density profiles change upon bending.

In order to aid the understanding of the results, we provide the relation between the curvature of the bicelle and the topology (concave or convex) of its inner and outer monolayers in Table 1.

**Table 1:** Relation between the curvature of the bicelle and the topology (concave or convex) of its inner and outer monolayers. Code names of the lipids in each monolayer are indicated.

Curvature, nm^−1^	0.2	0	−0.2
Outer monolayer (PC1, SM1, PE1)	Convex	Flat	Concave
Inner monolayer (PC2, SM2, PE2, PS2)	Concave	Flat	Convex

**Figure 2:**
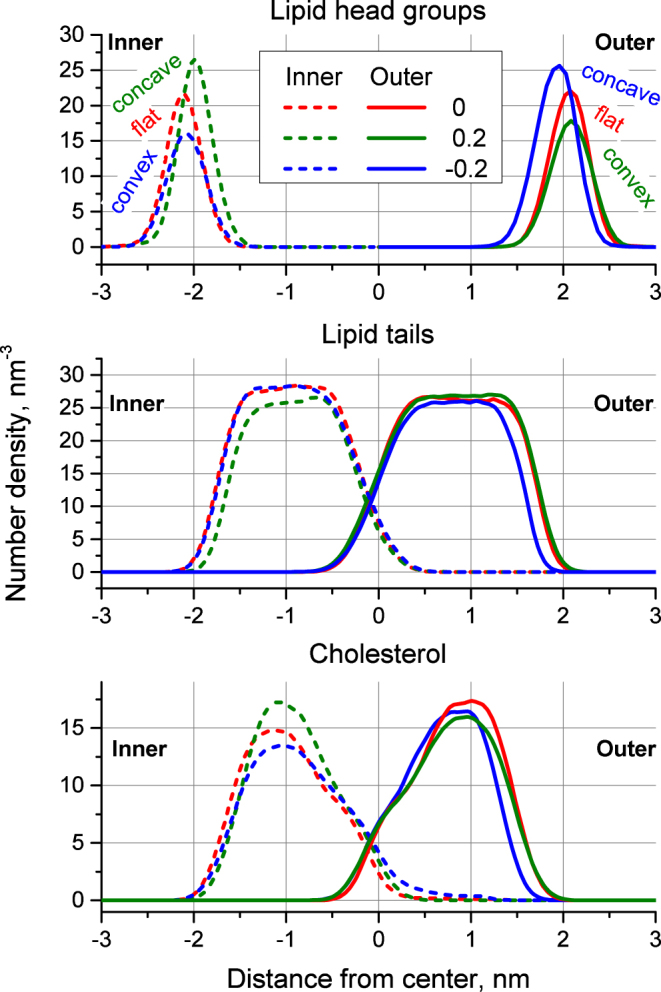
Density of lipid head groups, lipid tails and cholesterol of inner and outer monolayers of the bicelles with different curvatures. Topology of the monolayer, which corresponds to each curve (convex, concave or flat), is indicated in top panel.

Figure 2 shows that the curvature leads to visible changes in the density profiles. In particular it leads to a significant decrease in the density of lipid head groups on the convex membrane surface whilst it leads to an increase in their density on the concave surface (Fig. 2 top panel). The peak, which corresponds to the concave surface shifts closer to the membrane center by ~1.3 Å. At the same time, the peak corresponding to the convex surface does not move.

The edge of the tail distribution (the boundary of the hydrophobic region) of the concave monolayer also shifts towards the center of the membrane by ~1.2–1.3 Å while the tails of the convex monolayer are not affected. In the case of *c* = 0.2 there is also a visible depletion in the density of the tails in the inner concave monolayer (Fig. 2 middle panel).

The general shape of cholesterol distribution is similar in both flat and curved membranes (Fig. 2 bottom panel). The density of cholesterol in the inner monolayer exhibits the same behavior as that of the head groups: it increases for *c* = 0.2 and decreases for *c* = −0.2. The density in the outer monolayer changes in a less systematic way but clearly follows a decrease in the thickness of this monolayer for *c* = −0.2. The outer monolayer has a much higher content of SM which is known to form strong complexes with cholesterol. Due to this interaction, changes in the density of cholesterol in the outer monolayer could be less pronounced.

The results obtained show marked asymmetric behavior of the concave and convex monolayers. The density profiles of convex monolayers are barely affected by curvature while the concave monolayer becomes thinner by ~1.3 Å due to decrease in the thickness of its hydrophobic region. This effect is unlikely to be easily detectable experimentally due to the lack of resolution of the experimental techniques and small magnitude of the effect (about the lengths of one C-C bond in lipid tails). However, it may lead to changes in the macroscopic properties of the curved membranes, such as the permeability for hydrophilic compounds.

### Order parameter

The order parameter of the lipid tails is an important criterion that characterizes the lipid packing in the membrane. It shows the orientation and the general ordering of the hydrocarbon tails for particular depth in each membrane leaflet. The order parameter in flat bilayers could be determined experimentally and compared directly to simulation results. However, it is not clear how to determine this experimentally in curved membranes since the curvature is rapidly evolving as a local property. Thus, numerical simulations remain the only technique to estimate the changes of lipid ordering in curved membranes. Our results suggest that the order parameter of lipid tails changes significantly and non-trivially upon membrane bending.

**Figure 3:**
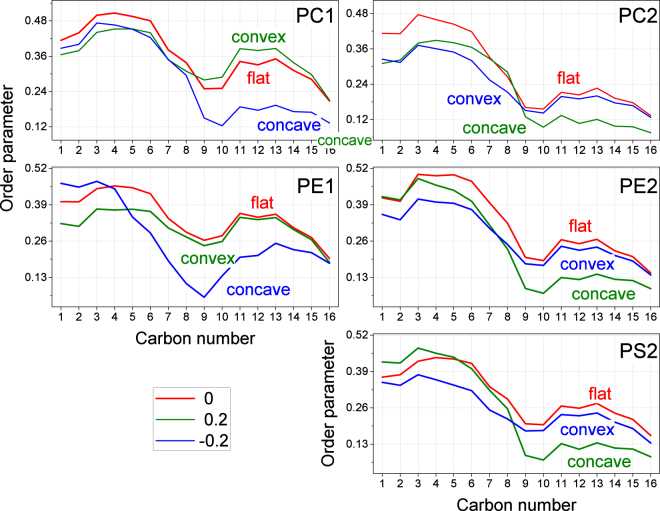
Order parameter of the lipid tails for all studied phospholipid species in inner and outer monolayer as a function of curvature. Each panel shows order parameter of indicated lipid in outer (1) or inner (2) monolayer. Topology of the monolayer, which corresponds to each curve (convex, concave or flat), is indicated.

Figure 3 shows the order parameter of the tails of all phospholipid species in both the inner and outer monolayers for different values of curvature. It can be clearly seen that the distal parts of the tails (below the double bond for carbons 9–10) of all phospholipids become much less ordered in concave monolayers, while keeping approximately the same ordering as flat membranes in convex monolayers. This result is expectable from the purely geometrical point of view. Indeed, in the concave monolayers the lipid head groups are “congested” while the volume available to the distal parts of the tails is larger than in the flat membrane. As a result distal parts of the tails fill available space by becoming less ordered.

The proximal parts of the tails (atoms between the head and the double bond) show lipid-specific behaviour. The proximal parts of PC tails are less ordered in curved membranes in comparison to the flat one but there is almost no difference between the convex and concave monolayers. The proximal parts of PE and PS tails in concave monolayers are ordered equally or even higher than in the flat membrane. In contrast, the proximal parts of PE and PS tails in convex monolayers are much less ordered than in the flat membrane. These results are not easily explainable by simple geometric considerations because the chemical nature of the lipid head groups becomes important for structuring proximal parts of the tails.

**Figure 4:**
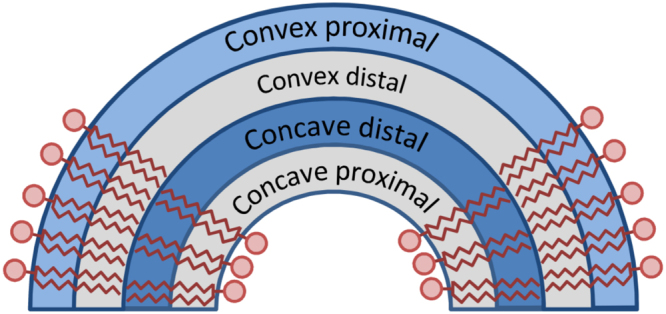
Scheme of the effect of curvature on the ordering of lipid tails. Proximal and distal parts of concave and convex monolayers are marked. The parts which show no significant change of ordering upon bending are shown in light gray. The part which shows a moderate decrease of ordering (convex proximal, “–” in Table 2) is shown in light blue. The part which show a significant decrease of ordering (concave distal, “--” in Table 2) is shown in blue.

Table 2 summarizes the observed effects of the curvature. Curvature never leads to a significant increase in the ordering while a dramatic decrease of ordering occurs in the distal parts of lipid tails in concave monolayers. A smaller but still significant decrease of ordering is observed in the proximal parts of the tails in a convex monolayer. The scheme in Fig. 4 illustrates this effect. The areas of lipid tails where there is a significant decrease of ordering are marked.

**Table 2:** Summary of the influence of curvature on the order parameter of phospholipids. The symbols show the change of ordering of proximal and distal parts of lipid tails in comparison to flat membrane. “−” means moderate decrease of ordering, “--’’ strong decrease of ordering, “− = ” slight decrease or no change, “ + = ” slight increase or no change.

Monolayer curvature	Convex		Concave	
Part of the tail	Proximal	Distal	Proximal	Distal
PC	-	+ /=	−	--
PE	-	−/=	+ /=	--
PS	-	−/=	+ /=	--

The changes of ordering in saturated sphingomyelin tails are different and should be analyzed separately. The most ordered part of SM tails in the flat membrane is in the region of carbons 7–8, while the proximal and distal parts of the tails are much less ordered. This maximum shifts to carbons 6–7 in the convex monolayers and to carbons 8–9 in the concave monolayers. Surprisingly, in the case of *c* = −0.2 the ordering of SM tails in both monolayers decreases significantly for all carbon atoms. In the case of *c* = 0.2 such a decrease is observed in the proximal part of the concave inner monolayer and in the distal part of convex outer monolayer (see Supplementary Fig. S2). The reason of such inconsistent behaviour is not clear.

An observed non-trivial decrease in the lipid order parameter in certain parts of the curved membrane could also be detected directly in specifically designed experiments. For example the ordering-sensitive fluorescent dyes with the chromophores located at a well-defined depth may allow us to observe the curvature-related decrease of ordering directly. This effect may also influence the permeability of the membrane to bulky compounds, which may disturb the ordering of surrounding lipid tails.

### Area per lipid

**Figure 5:**
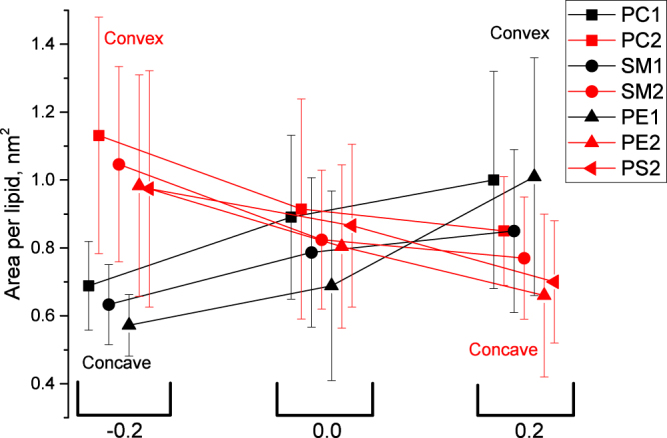
Average areas per lipid for all lipid species of inner and outer monolayers as a function of curvature.

The area per lipid is one of the basic properties used to characterize the lipid bilayers. It is known to depend on the mechanical strain of the membrane, lipid composition and cholesterol content. Thus, it is expected to depend strongly on the membrane curvature as well. Our methodology allows us to determine the area per each lipid in each simulation frame using the Voronoi tessellation technique. This provides reliable estimates of the average area per lipid for all lipid species in inner and outer monolayers (Fig. 5). All lipid species follow the same trend, namely a decrease of the area per lipid from convex to flat to concave monolayers. This decrease is remarkably large and reaches between 25–35% depending on the lipid type. The areas per lipid of the same lipid species in the inner and outer monolayers are different but large standard deviations make the significance of these differences less reliable.

The observed dramatic changes in the areas per lipid could be explained by geometrical considerations. Indeed, the head groups of the lipids become congested in the concave monolayers while much more space is available to them in the convex monolayers. As a result the area per lipid is expected to be maximal in convex monolayers and minimal in the concave ones, which is in perfect agreement with the trends in Fig. 5.

The contribution of the lateral mechanical strain to the changes of the areas per lipid is minimal in our setup. The inner and outer monolayers equilibrate their surface areas and volumes quickly due to the bicelle caps, which serve as adjustable reservoirs for extra lipids. Supplementary Table S1, available online, shows the mean number of lipids found in the inner and outer monolayers of the bicelle for different curvature. Only the central part of the bicelle, which is used for analysis and excludes the caps, is considered. It is clearly seen that the number of lipids in both monolayers change in accordance with the membrane curvature. The caps of the bicelle accommodate excessive lipids from the concave monolayer and donate necessary amount of lipids to the convex monolayer which prevents development of mechanical strain effectively.

### Cholesterol distribution and orientation

Cholesterol molecules are known to be highly dynamic in the lipid bilayer. They diffuse quickly in the lateral direction, change position vertically in the lipid monolayers and exhibit flip-flop transitions within the time scale of hundreds of nanoseconds. The inclination angle of cholesterol may also change following the topology of lipid tails, which surround rigid sterol rings.

The inclination angle was computed as an angle between long axis of cholesterol molecule and the local outer normal of the corresponding membrane monolayer (see Methods for details). We computed inclination angles of cholesterol molecules and distribution of their distances from the membrane surface for both inner and outer monolayers as a function of curvature. Two distinct fractions of cholesterol molecules were detected. The major fraction is intercalated between the lipid tails with a vertical orientation (roughly parallel to the membrane normal). The minor fraction is located in the center of the hydrophobic core of the membrane and is oriented horizontally (perpendicular to the membrane normal). The existence of these two fractions was shown before in coarse-grained^15^ simulations, however, their dependence on the membrane curvature was never previously studied.

**Figure 6:**
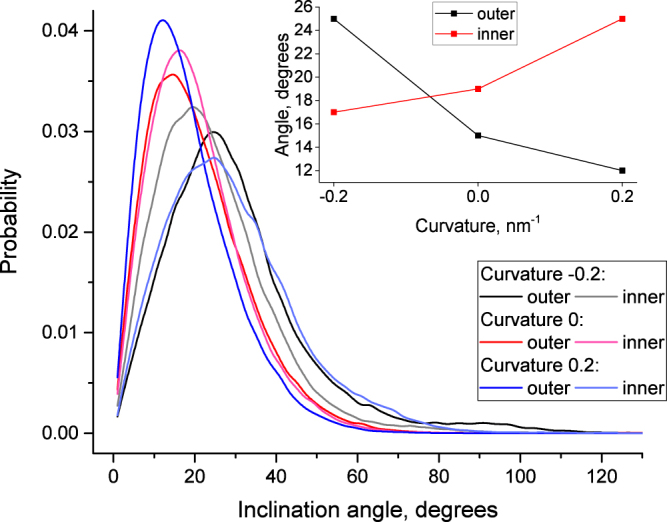
Distribution of inclination angles of cholesterol molecules in inner and outer monolayers as a function of the membrane curvature. The inset shows the position of the major maximum of cholesterol distribution as a function of curvature.

Figure 6 shows the distribution of the cholesterol inclination angle depending on the curvature and the monolayer. The main maximum corresponds to the major cholesterol fraction while the minor fraction is visible as a residual probability density around 90°. The position of the major peak shows a pronounced dependence on the curvature. The inset in Fig. 6 shows that the inclination angle of the cholesterol decreases when the curvature of a particular monolayer changes from concave to flat or to convex. This decrease is equally pronounced for both monolayers. The absolute value of the inclination angle is systematically larger in the inner monolayer. This may be caused by the influence of high SM concentration in the outer monolayer which aligns cholesterol molecules to the membrane normal and decreases the inclination angle.

**Figure 7:**
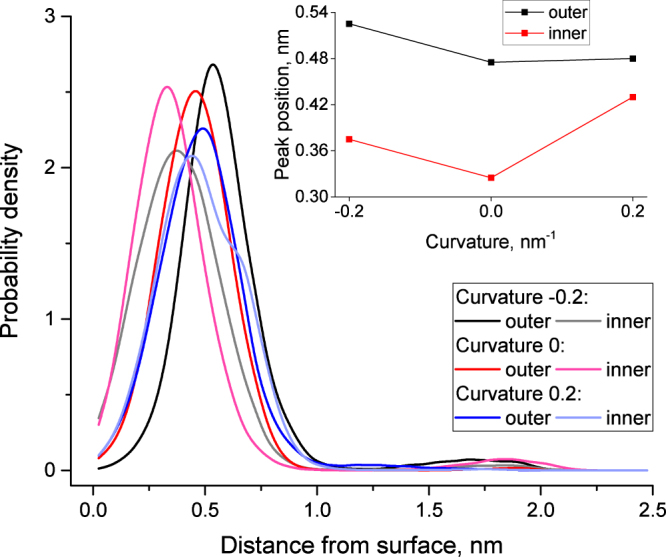
Distribution of the distances of cholesterol molecules from the membrane surface in inner and outer monolayers as a function of the membrane curvature. The distance is measured from approximated membrane surface to the OH group of cholesterol. The inset shows the position of the major maximum of cholesterol distribution as a function of curvature.

Figure 7 shows the distribution of the depth of cholesterol location in the membrane depending on curvature and the monolayer. The minor cholesterol fraction is clearly visible in this figure as a small peak near ~1.75 nm. The position of the peak which corresponds to the major cholesterol fraction changes significantly depending on the curvature. The distance of cholesterol head groups from the membrane surface is minimal in the flat membrane and increases upon bending (inset in Fig. 7). This effect is less pronounced in the outer monolayer. Regardless of the curvature it is also evident that cholesterol molecules are located deeper in the inner monolayer than that of the outer monolayer. This effect is likely to be caused by the complexation of cholesterol with SM lipids in the outer monolayer which results in its lower vertical mobility.

The minor cholesterol fraction resides in the central region of the membrane, thus it cannot be reliably attributed to either the inner or outer monolayer. It should be considered as a single pool of cholesterol molecules located between the membrane leaflets. The strong dependence of the relative abundance of the minor cholesterol fraction (relative to total number of cholesterol molecules in the system) on curvature in observed (see Supplementary Fig. S3). In the membrane with positive curvature (*c* = 0.2) there are no cholesterol molecules in the minor fraction between the leaflets. In the flat membrane (*c* = 0) about 1.7% of cholesterol is in the minor fraction. Finally, in the membrane with a negative curvature (*c* = −0.2) the minor fraction increases to 2.5%. This effect is again in good agreement with higher SM concentration in outer monolayer. Indeed, for *c* = −0.2 the outer concave monolayer is compressed at the level of head groups. Rigid complexes of cholesterol with SM are likely to become less energetically favorable in such conditions and some cholesterol molecules are expelled towards the center of the membrane, which helps to relax the conformational strain of SM lipids. In contrast for *c* = 0.2 the outer monolayer is convex. This creates some extra free volume for the cholesterol molecules between the rigid saturated tails of SM lipids, and the minor cholesterol fraction disappears.

Our results are in line with previous observations showing the high mobility of cholesterol and sensitivity of its position and orientation to the state of surrounding lipids. In addition to the well-known effects of the lipid content and mechanical strain, the depth and inclination of cholesterol molecules in the membrane appears to be highly sensitive to the curvature of individual monolayers.

### Limitations

Our simulations have several limitations which need to be mentioned. The major limitation of our technique is that of maintaining the membrane curvature by the external walls, due to the mechanical restraints imposed by the walls. In the case of the flat membranes it is possible to compare the bicelle with the walls with a free lipid bilayer with periodic boundary conditions. We show that the influence of the walls is minor in this case and could be neglected. However, no such control is possible for the curved membranes. Strictly speaking it is not known if the influence of the walls remains as small for the curved bicelles as it was in the flat membrane. That is why the effects of curvature revealed in this work should be interpreted with caution. It is very unlikely that the effects themselves are artifacts produced by the simulation setup, but their absolute values could be influenced by the walls and thus should be interpreted at the semi-qualitative level.

Another limitation comes from the fact that such complex multicomponent system as a model plasma membrane is unlikely to be equilibrated completely at sub-microsecond time scales. Ideal sampling of such heterogeneous membrane may only be reached at larger times than the time of lateral diffusion of lipids through the whole bicelle (tens or even hundreds of microseconds). Unfortunately, such times are not reachable in all atom simulations providing limited access to computational resources, which is a common limitation of all MD studies in the field. Moreover, SM-rich outer monolayer is likely to exhibit spontaneous demixing of the lipid species and the formation of raft-like complexes of cholesterol and SM at the microsecond time scales. No such events are sampled in our simulations. Despite limited sampling, our simulations revealed reliable differences in behavior of different lipid species which are likely to stand at larger time scales as well.

### Perspectives

Our technique of maintaining a desired curvature of the membrane allows computing any macroscopic or mesoscopic property of the membrane as a function of curvature. In particular it allows us to compute the potentials of mean force (PMFs) of translocation of various compounds through the curved membranes. Such simulations are not possible without fixing the global membrane shape because the sampling of the PMF windows should be performed for a predefined value of the curvature. Comparison of the PMFs of different compounds in flat and curved membranes will be performed in our future work. Another line of research will be the comparison of normal asymmetric membranes with the membranes of cancer and apoptotic cells where the lipid asymmetry disappears completely or partially. The data on the influence of curvature on such membranes could be especially interesting in combination with the PMFs for various drugs and membranotropic agents.

## Methods

### Simulation details

All simulations were performed using GROMACS 5.1.2 software^39^. The Slipids force field^40^ was used for lipids in combination with the AMBER99sb force field for water and ions. All simulations were performed in NPT conditions using the Berendsen barostat^41^ at 1 atm and velocity rescale thermostat^42^ at 320 K if not stated otherwise. An integration step of 2 fs with the group cutoff scheme was used^43^. All bonds were treated as rigid constraints. Long range electrostatics was computed with the PME method^44^. Preparation of the systems and data analysis was performed using the Pteros 2.0 molecular modeling library^45,46^. VMD 1.9.2^47^ was used for visualization.

### Preparation of the membrane models

In this work we used the model of plasma membrane of mammalian cells with asymmetric lipid content in the outer and inner monolayers. The membrane was prepared as a cylindrical bicelle which is limited by semi-circular caps in XZ plain and is infinite in the Y direction. Such a shape is necessary for producing curved membranes as described below. Indeed, when the curvature is imposed on the membrane the areas of concave and convex monolayers become different and the lipids have to redistribute in order to compensate for the resulting mechanical strain. There are several ways of achieving this in MD simulations. The first is to use an “inverted domains” setup which was used in our previous studies on the spontaneous bending of asymmetric membranes^15^. This method is not optimal for the current work because it doubles the size of the system and makes it less computationally friendly. The second method requires the converting of a bilayer to a bicelle where the caps can serve as reservoirs for lipid redistribution upon bending. This setup was recently used with great success for the coarse-grained curved membranes^48^ and was chosen for this study. The reader is referred to^48^ for a detailed description and discussion of this setup.

The lipid content of inner and outer monolayers is different and their mixing in the regions of the bicelle caps should be prevented. In order to achieve this we used the method of selective artificial repulsive potentials similar to the one used in our previous works^15,37,48^. All heavy atoms of the lipids (except the carbon atoms of the distal parts of the tails below the double bond) were assigned an additional weak Van der Waals repulsive potential (*σ* = 0.8 nm, *ε* = 1·10^−7^ J/mol) which acts between the lipids of inner and outer monolayers only. This allows the ends of the lipid tails to interact normally in the bilayer region of the bicelle while the lateral contacts between the lipids from the different monolayers in the caps of the bicelle become energetically unfavorable. Such a setup prevents the mixing of the monolayers effectively without any detectable effect on the bilayer part of the system. Cholesterol molecules can diffuse freely through the caps of the bicelle which facilitates their optimal distribution between the monolayers. This setup is illustrated by Supplementary Figure S4 available.

Table 3 shows the lipid content of the monolayers which was designed according to well-established lipid content of mammalian erythrocyte membranes^49^. Phosphatidylinositol was not included into the present simulations due to its small concentration which results in about one molecule per system.

**Table 3:** Lipid content (absolute number of molecules) of the monolayers of simulated membrane. Initial cholesterol distribution is shown.

Component	Outer monolayer	Inner monolayer
SM (sphingomyelin)	42	12
PC (1,2-dioleoyl-sn-glycero-3-phosphocholine)	46	14
PE (1,2-dioleoyl-sn-glycero-3-phosphoethanolamine)	14	46
PS (1,2-dioleoyl-sn-glycero-3-phospho-L-serine)	0	30
Cholesterol	51	51

Initially each monolayer contains 102 lipids and 51 cholesterol molecules. Cholesterol molecules are allowed to diffuse through the bicelle caps and redistribute themselves between the monolayers during the simulations. The lipids are arranged initially into a 17 × 6 grid in each monolayer with cholesterol molecules intercalated randomly between them. The system is pre-equilibrated as an infinite bilayer with periodic boundary conditions for 10 ns and then converted to a bicelle by adding extra layers of water from both sides in the X direction. After the addition of the restricting walls (see below), the system is equilibrated as a planar bicelle for ~500 ns.

The preparation of pure 1,2-dioleoyl-sn-glycero-3-phosphocholine (PC) membrane, which was used for additional validation of the minimal influence of the walls on the membranes with different lipid content, is described in Supplementary Information.

### The methodology of membrane bending

In this work we developed a method for keeping global membrane curvature at a given value by shaping the membrane by means of artificial dummy particles. The idea is to put the membrane between two repulsive surfaces of artificial particles - the walls - which restrict the global shape of membrane but which do not affect lateral dynamics of individual membrane lipids. In this work we bend the membrane in a single XZ plane for simplicity, however, our method is applicable to any desired shape in three dimensions.

The walls are composed of independent non-interacting beads which consist of an anchor and shell particles. The anchor is a dummy particle which is fixed in absolute coordinates and which does not interact with any other particles in the system. The shell particle is connected to an anchor by a harmonic bond of zero length with the force constant of 10 kJ·mol^−1^·nm^−2^. The shell particles selectively interact with the carbon atoms of the lipid acyl tails by Van der Waals interactions only. Parameters of this Lennard-Jones interaction are *σ* = 0.85 nm and *ε* = 1·10^−5^ J/mol which means that the potential is purely repulsive within the short-range cut-off *r*_*c*_ = 0.8 nm adopted in Amber force field^50^. Such a setup eliminates unwanted direct interactions of the lipid atoms with fixed anchor particles and allows the walls to be positioned precisely at the same time by moving anchors to the desired position.

The walls are positioned initially at the level of lipid head groups of each monolayer of the flat bilayer at a distance of 5 nm from each other. The dummy beads are arranged in a rectangular grid in the plane of the wall with a spacing of ~0.51 nm.

The shell particles of the walls are impermeable for the hydrophobic lipid tails but completely transparent for all other atoms. Thus, they restrict the general shape of the membrane hydrophobic core effectively without influencing the lateral diffusion of the lipids or the dynamics of lipid head groups, cholesterol, ions and water molecules. The distance between the walls and the parameters of repulsive Van der Waals interactions are adjusted empirically in order to minimize the influence of the walls on the membrane structure and properties. The density profiles of various chemical groups of the lipids were compared for planar bilayers with and without the walls. The results obtained (presented in the Results section) show that the changes in the structure of the bilayer introduced by the walls are barely visible and can be safely neglected.

The procedure of bending the membrane starts from the planar bicelle equilibrated in the presence of the walls. The shape of the membrane is changed gradually by moving the anchor particles of the walls towards their new locations, which correspond to the membrane with desired curvature. The shell particles push the hydrophobic core of the membrane and force it to bend accordingly. The technical details of this procedure are available online in Supplementary Information.

**Figure 8:**
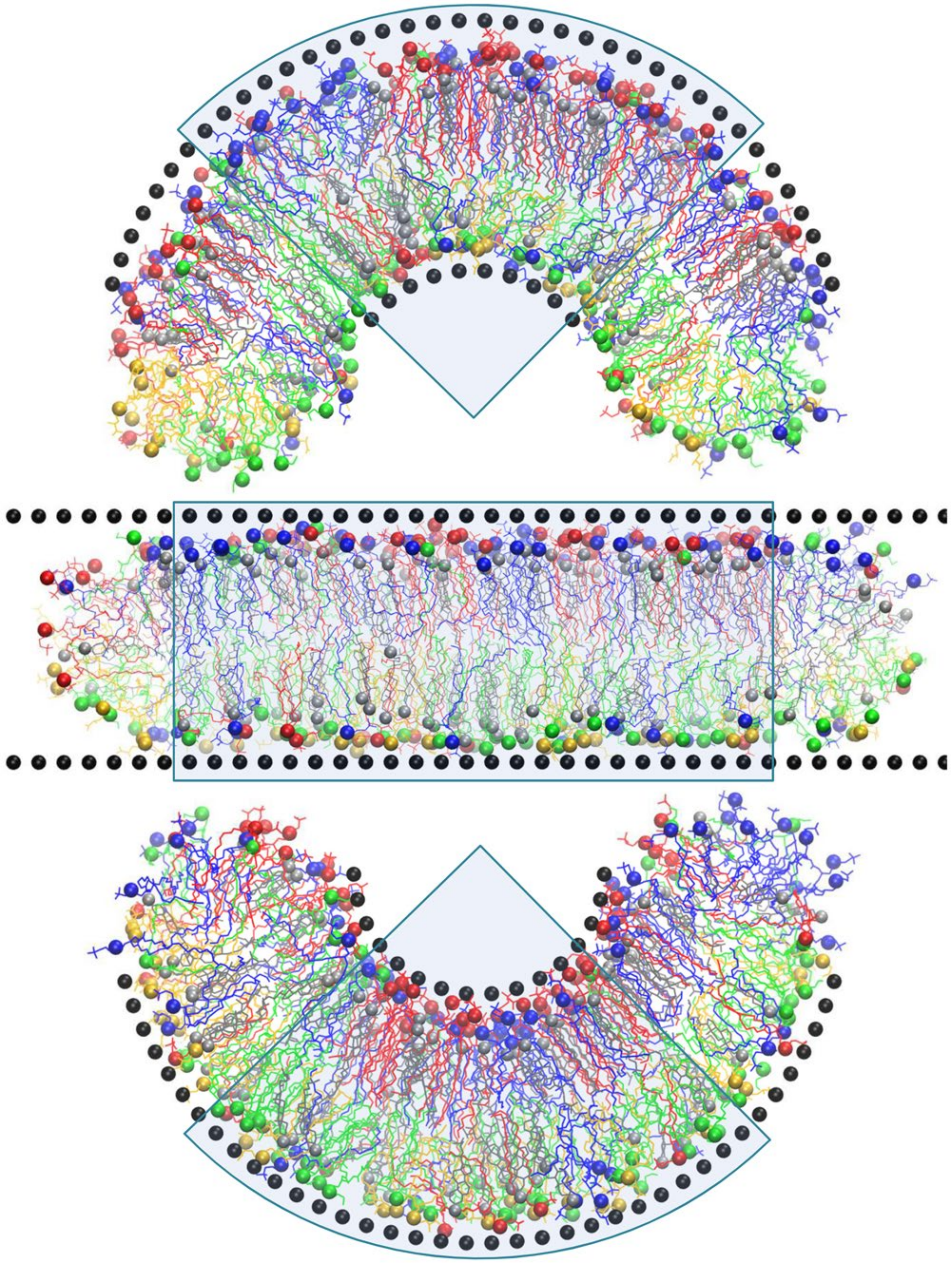
Snapshots of the simulated systems with the curvature c = 0.2 nm^−1^ (top), c = 0 nm^−1^ (middle) and c = −0.2 nm^−1^ (bottom). The wall particles are shown as black spheres. PC is blue, SM is red, PE is green and PS is yellow. Cholesterol molecules are gray. Head groups of lipids and cholesterol are shown as spheres. The sectors which are used for analysis are shaded.

Figure 8 shows three production systems used in this work: the initial planar system with zero curvature (*c* = 0 nm^−1^, middle panel in Fig. 8), the system with positive curvature (*c* = 0.2 nm^−1^, top panel in Fig. 8) and the system with negative curvature (*c* = −0.2 nm^−1^, bottom panel in Fig. 8). All systems were subject to production runs which were 250 ns in duration.

### Determining the area per lipid and cholesterol inclination angle

The areas per lipid were determined using the Voronoi tessellation as described in our previous work^51^. In brief, the tangent membrane plane was determined in the vicinity of the head group of each lipid. Positions of adjacent lipid heads are projected onto this plane and the area of the Voronoi polyhedron centered at the target lipid is computed. This technique allows us to compute the areas per lipid and to obtain statistics per lipid type.

The cholesterol inclination angle and position are computed as described in our previous work^51^. The angle is computed relative to the local membrane using the normal position of the cholesterol -OH group. The long axis of the cholesterol molecule is defined as a vector from the carbon atom at tail branching to -OH group.

## Conclusions

In this work we described a technique which allows the simulations of realistic membranes with asymmetric lipid content of monolayers at a predefined value of curvature. Our work provides an insight into the changes of various properties of the plasma membrane upon bending. The major results could be summarized as follows:

- The thickness of the hydrophobic core of the concave monolayer decreases by approximately 1.3 Å in comparison to that of the flat membrane, while the thickness of the convex monolayer does not change. This effect is almost symmetric in both the inner and outer monolayers.
- The area per lipid increases in the convex monolayer and decreases in the concave monolayer in comparison to that of the flat membrane. As a result the density of the head groups decreases in the convex monolayer and increases in the concave monolayer. This effect is pronounced to different extent for inner and outer monolayers
- The order parameter of the phospholipids decreases significantly in the distal parts of the tails in the concave monolayer and to a lesser extent in the proximal part of the tails of convex monolayer.
- The cholesterol inclination angle decreases when the curvature of a particular monolayer changes from concave to convex. The magnitude of this effect is larger in the outer monolayer.
- The distance of the cholesterol heads from the membrane surface is minimal in the flat membrane and increases upon bending. The magnitude of this effect is larger in the inner monolayer.
- The amount of cholesterol in the minor fraction located between the membrane leaflets is zero in the membrane with positive curvature and increases to 1.7% in the flat membrane and to 2.5% in the membrane with negative curvature.

It is possible to conclude that our technique of fixing the membrane curvature provides a robust way of studying various properties of the curved membranes, which are otherwise impossible to detect due to the transient nature of the membrane curvature in free MD simulations.

## Electronic supplementary material

Supplementary information
