# Supplementary material for: The influence of curvature on the properties of the plasma membrane. Insights from atomistic molecular dynamics simulations
Source: Sci Rep. 2017 Nov 22;7:16078. doi: 10.1038/s41598-017-16450-x (PMC5700167; doi:10.1038/s41598-017-16450-x)
Supplement: Supplementary file 1 — Supplementary information [file 41598_2017_16450_MOESM1_ESM.pdf]

The influence of curvature on the properties of the plasma membrane. Insights from atomistic molecular dynamics simulations.

Supplementary information

Semen O. Yesylevskyy, Timothée Rivel, Christophe Ramseyer

Preparation of pure PC membrane

The reference pure PC bilayer contained 128 DOPC lipids and 5120 water molecules. It was equilibrated in the same conditions and with the same parameters as the main asymmetric membrane described in the text. The only difference in the simulation setup was slightly smaller distance between the repulsive walls of dummy particles (~4.95 nm in pure PC system vs. 5.0 nm in the plasma membrane system). This difference resulted in slightly increased influence of the walls on the hydrophobic core of the membrane, which is visible as a small decrease of the thickness of the hydrophobic core in Fig. S1 in the presence of the walls. This influence was eliminated completely in the production setup for the plasma membrane by increasing the distance between the walls to 5 nm.

The flat bicelle with the walls was built separately using the procedure described in Methods. It contained 288 lipids and ~46000 water molecules. Due to the absence of any difference between the monolayer the repulsive potential for preventing their mixing in the bicelle cups was not used. Both systems were equilibrated for 100 ns. After that the 100 ns production runs were performed.

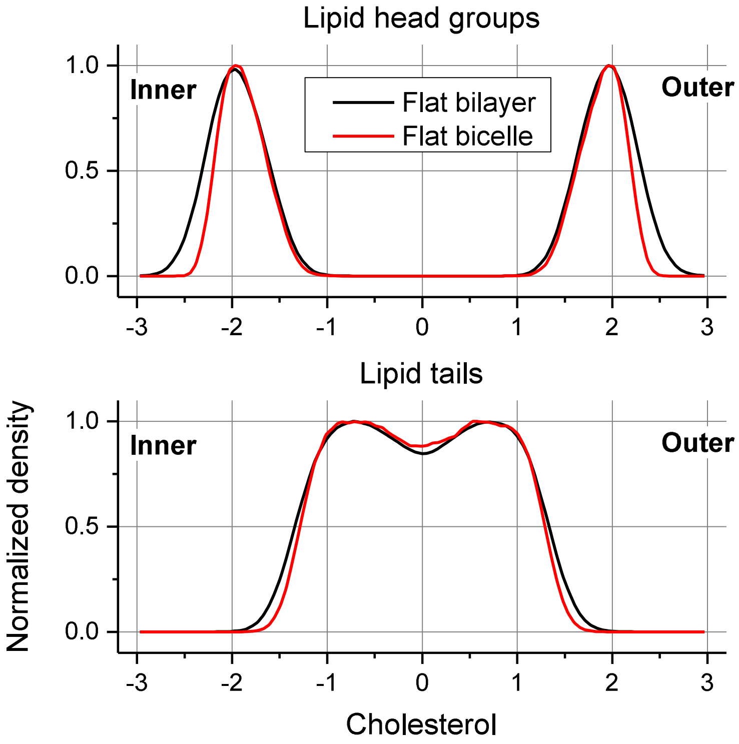

Figure S1. Normalized densities of lipid head groups and tails in reference flat bilayer and flat bicelle for symmetric pure PC membrane.

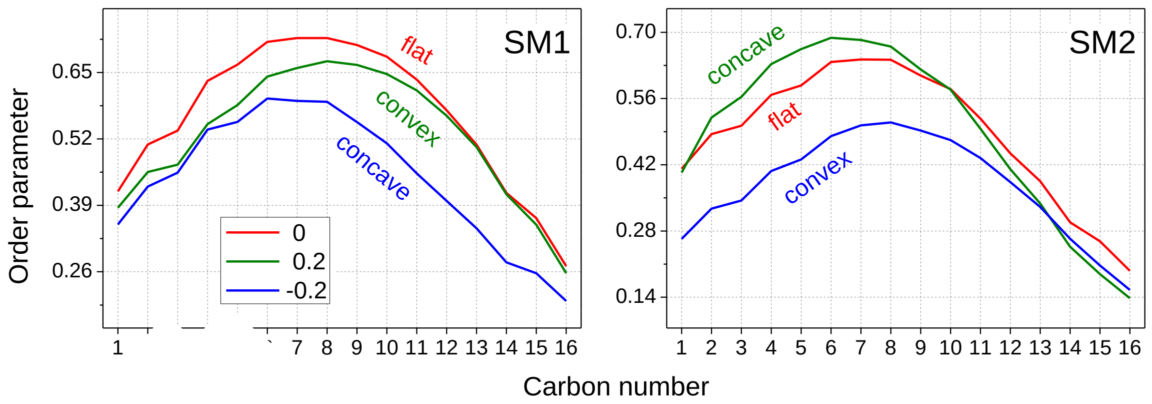

Figure S2. Order parameter of the sphingomyelin tails in inner (SM1) and outer (SM2) monolayers as a function of curvature. Topology of the monolayer, which corresponds to each curve (convex, concave or flat), is indicated.

Table S1. Mean number of lipids in the inner and outer monolayers of the bicelle as a function of the membrane curvature. The central part of the bicelle, which is used for analysis and does not include the caps, is considered.

Curvature, nm^{-1}	-0.2	0	0.2
Outer monolayer	36.7 (concave)	40.6 (flat)	53.1 (convex)
Inner monolayer	50.8 (convex)	39.1 (flat)	33.4 (concave)

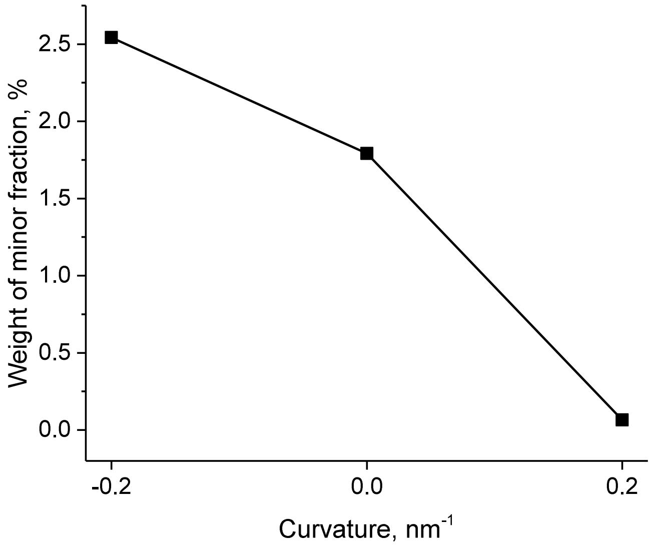

Figure S3. Abundance of the minor cholesterol fraction located between the membrane leaflets as a function of curvature.

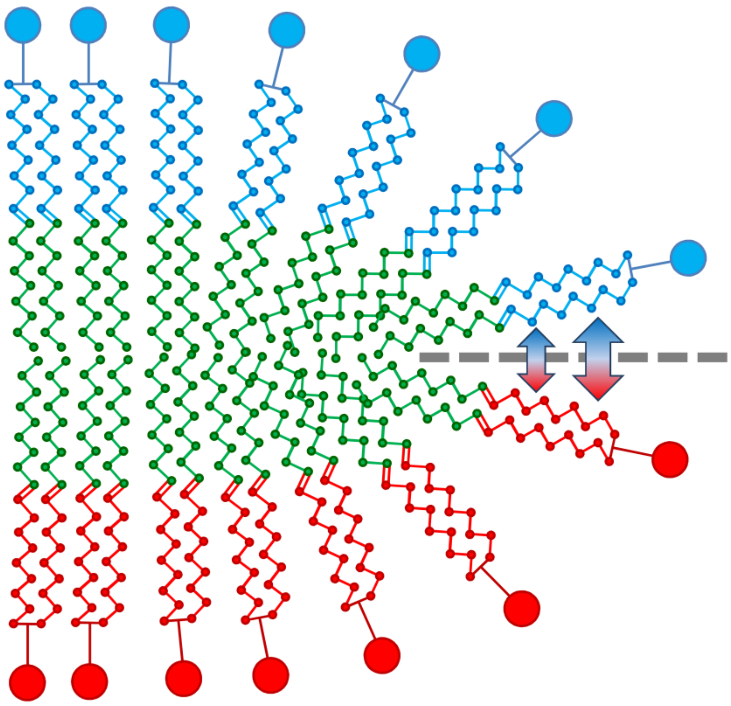

Figure S4. Scheme of the bicelle cap which prevents mixing of the lipids from different monolayers. Distal parts of the tails (below the double bond), which interact normally, are shown in green. Proximal parts of the tails and head groups of lipids from different monolayers are shown in blue and red respectively. There is an additional artificial Van der Waals repulsion between blue and red atoms (shown by the arrows) which prevents mixing of the lipids from different monolayers. Approximate boundary between the monolayers is shown by gray dashed line.

Membrane bending procedure

In order to produce the curved membranes the following procedure is used. The planar bilayer equilibrated in the presence of the walls is used as initial system. The mean curvature *c* which we wanted to obtain is set and the center of the curvature is determined as shown in Figure S5. New positions of the anchor particles, which correspond to desired curvature, are also computed. The distances between the dummy beads within each wall are kept constant to ensure that the density of dummy particles per unit area of each monolayer is the same as in the planar bilayer. After that all anchor particles are moved with a constant rate towards their new positions during a time period of 5 ns. The dummy particles push the hydrophobic core of the membrane and force it to bend accordingly. Due to the different arc lengths of upper and lower walls some beads of the latter, which appear beyond the needed sector, are removed as shown in Figure S5, The system is then equilibrated for 200 ns with the anchor particles fixed in their new positions in order to relax any strain introduced by the forced bending. The bending is performed in several stages with a curvature step of 0.05 nm^{-1} (from 0 to 0.05 nm^{-1}, from 0.05 to 0.1 nm^{-1}, *etc.*) to reduce the mechanical stress imposed on the bicelle at each stage. For our asymmetric bicelle, the direction of the bending (upwards or downwards) is important due to the different lipid content of the

monolayers. Nine systems were generated with curvatures of -0.2, -0.15, -0.1, -0.05, 0, 0.05, 0.1, 0.15 and 0.2 nm^{-1} where positive curvature means bending downwards (inner monolayer is concave as shown in **Ошибка! Источник ссылки не найден.**) while negative curvature means bending upwards (outer monolayer is concave). The two systems with the largest curvatures (c = 0.2 nm^{-1}, top panel in **Ошибка! Источник ссылки не найден.**, and c = -0.2 nm^{-1}, bottom panel in **Ошибка! Источник ссылки не найден.**) were used for the production runs which were 250 ns in duration.

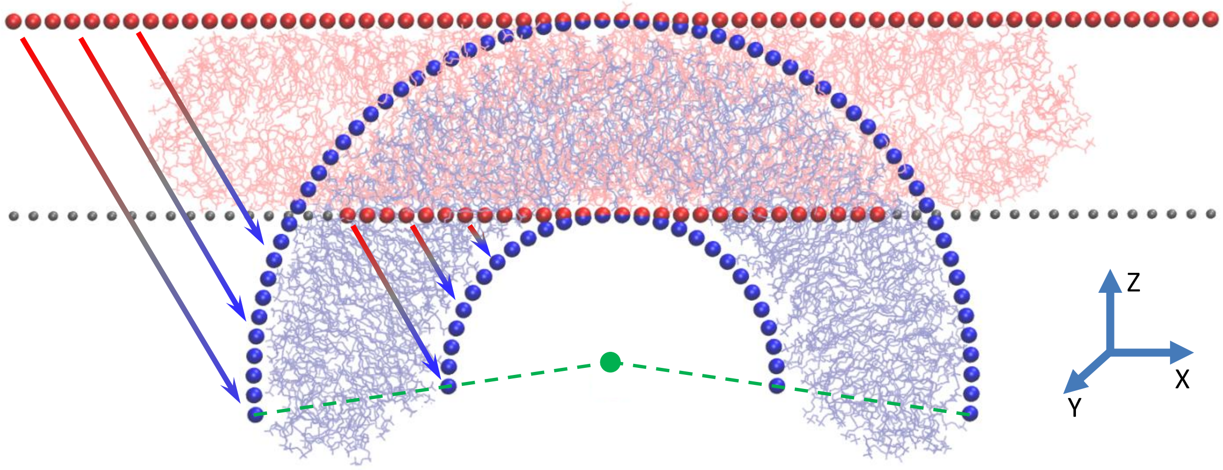

Figure S5. Scheme of membrane bending showing a model bicelle with a final curvature of 0.15 nm^{-1}. The initial planar bicelle is shown as pink lines. The final curved bicelle is light blue. Wall particles are shown as red (initial positions) and blue (final positions) spheres. The wall particles from lower wall, which are dropped after the bending, are gray. The arrows show the trajectories of pulling of the wall particles. Green point indicates the center of curvature and the dashed lines indicate the sector occupied by the walls.
